# An efficient deep learning model for tomato disease detection

**DOI:** 10.1186/s13007-024-01188-1

**Published:** 2024-05-09

**Authors:** Xuewei Wang, Jun Liu

**Affiliations:** Shandong Provincial University Laboratory for Protected Horticulture, Weifang University of Science and Technology, Weifang, China

**Keywords:** Greenhouse cultivation environment, Deep learning, Object detection, YOLO, Transformer, Tomato disease

## Abstract

Tomatoes possess significant nutritional and economic value. However, frequent diseases can detrimentally impact their quality and yield. Images of tomato diseases captured amidst intricate backgrounds are susceptible to environmental disturbances, presenting challenges in achieving precise detection and identification outcomes. This study focuses on tomato disease images within intricate settings, particularly emphasizing four prevalent diseases (late blight, gray leaf spot, brown rot, and leaf mold), alongside healthy tomatoes. It addresses challenges such as excessive interference, imprecise lesion localization for small targets, and heightened false-positive and false-negative rates in real-world tomato cultivation settings. To address these challenges, we introduce a novel method for tomato disease detection named TomatoDet. Initially, we devise a feature extraction module integrating Swin-DDETR’s self-attention mechanism to craft a backbone feature extraction network, enhancing the model’s capacity to capture details regarding small target diseases through self-attention. Subsequently, we incorporate the dynamic activation function Meta-ACON within the backbone network to further amplify the network’s ability to depict disease-related features. Finally, we propose an enhanced bidirectional weighted feature pyramid network (IBiFPN) for merging multi-scale features and feeding the feature maps extracted by the backbone network into the multi-scale feature fusion module. This enhancement elevates detection accuracy and effectively mitigates false positives and false negatives arising from overlapping and occluded disease targets within intricate backgrounds. Our approach demonstrates remarkable efficacy, achieving a mean Average Precision (mAP) of 92.3% on a curated dataset, marking an 8.7% point improvement over the baseline method. Additionally, it attains a detection speed of 46.6 frames per second (FPS), adeptly meeting the demands of agricultural scenarios.

## Introduction

According to a recent report released by the Food and Agriculture Organization of the United Nations, preliminary findings suggest that over one-third of annual agricultural production losses are caused by plant diseases [1]. Plant infectious diseases can lead to rapid spreading and enormous losses. Hence, early discovery and diagnosis of these diseases is crucial. In the past, agricultural experts performed plant disease detection, which required a high level of professional knowledge. However, this task was time-consuming, labor-intensive, and prone to error [2]. Traditional plant disease detection methods based on manually extracting features are complex and inefficient. The progress of artificial intelligence and computer vision technology, especially the development of deep learning, offers solutions to many problems in different fields, including agriculture, and produces more accurate results than traditional methods [3].

**Fig. 1:**
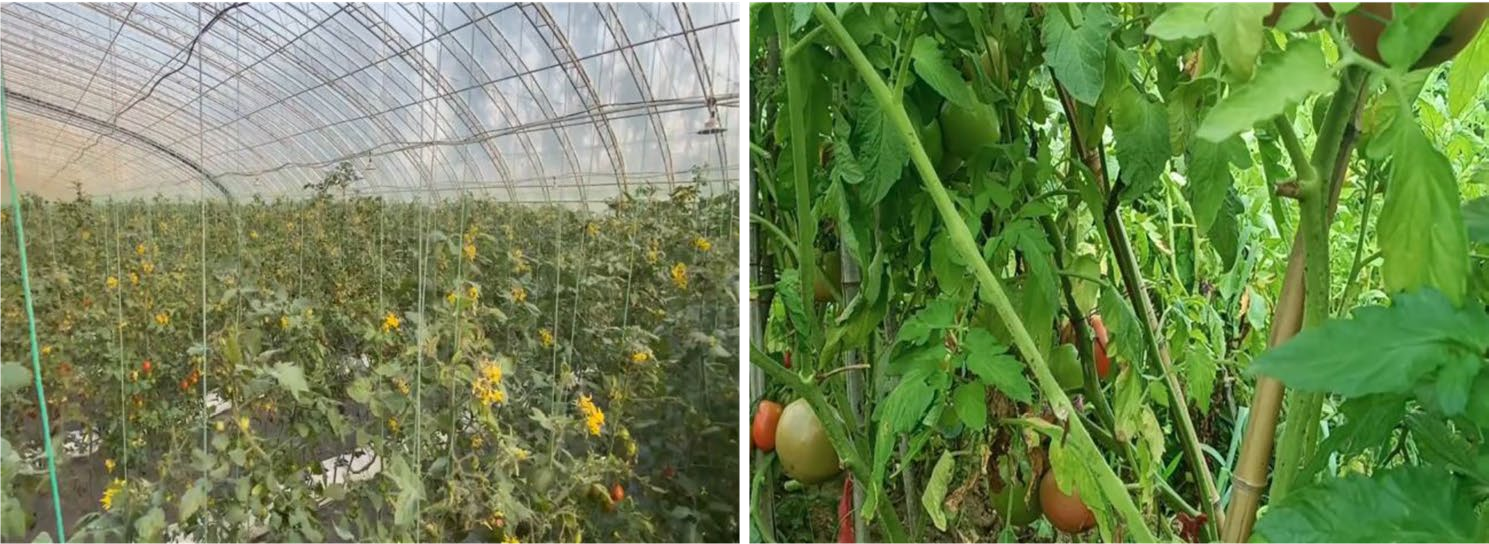
Tomato greenhouse growing

Using tomatoes as an example, they are widely cultivated worldwide [4], with significant acreage and yield (Fig. 1). Tomatoes not only boast a delightful taste but also contain a variety of essential micronutrients, rendering them highly nutritious and indispensable in daily diets [5]. Greenhouse cultivation provides an advantageous environment for year-round tomato production but also fosters conditions conducive to disease occurrence and development. However, the high temperature and humidity within the facilities often lead to the proliferation of various diseases, significantly impacting tomato yield and quality [6]. Greenhouse tomatoes are particularly susceptible to a multitude of rapidly spreading diseases, resulting in significant and persistent damage. During the winter and spring seasons, high temperatures, humidity, and weak light within greenhouses have contributed to widespread disease occurrence, resulting in poor growth and a serious impact on quality and yield [7].

**Fig. 2:**
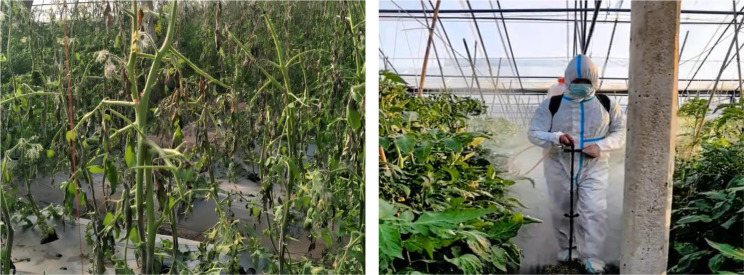
Tomato disease damage. **(a)** Widespread dissemination of diseases, **(b)** Extensive spraying of agricultural chemicals

In recent years, due to climate change exacerbating the probability of tomato diseases, extensive spraying of agricultural chemicals has ensued, which has resulted in significant damage and persistent high levels of agricultural chemical residues in tomatoes (Fig. 2).

**Fig. 3:**
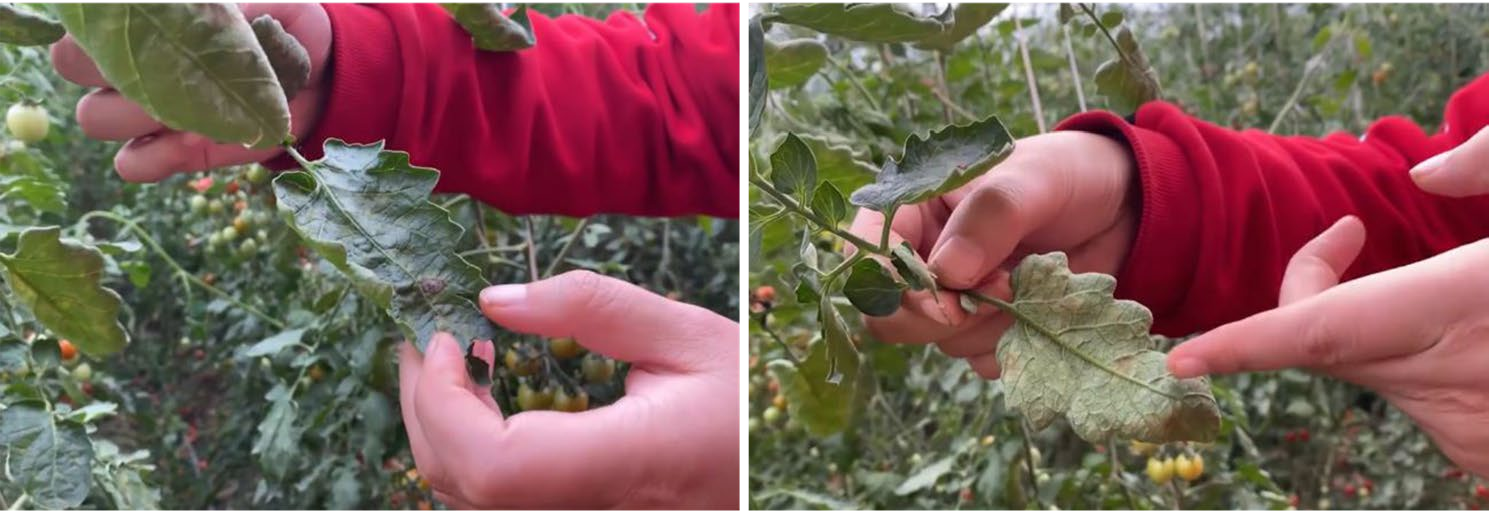
Identification of tomato diseases using empirical methods. **(a)** Diseases on the front surface of leaves, **(b)** Diseases on the back surface of leaves

This has caused serious food safety issues and significantly reduced the economic benefits of tomato cultivation. Consequently, rapid and accurate disease detection plays a crucial role in the prevention and control of tomato diseases [8, 9]. Currently, the identification and control of tomato diseases primarily rely on empirical methods (Fig. 3), which are characterized by low timeliness, poor accuracy, and high requirements for the professional skills of inspectors, often resulting in misdiagnosis and missed detections. Therefore, leveraging machine vision technology for precise detection of greenhouse tomato diseases has emerged as an urgent research topic.

Over the past few years, there has been an increasing inclination towards the application of artificial intelligence (AI) methodologies to various areas of agriculture including crop planting, harvesting, and disease detection [10, 11]. Nevertheless, further advancements are imperative in this realm.

**Fig. 4:**
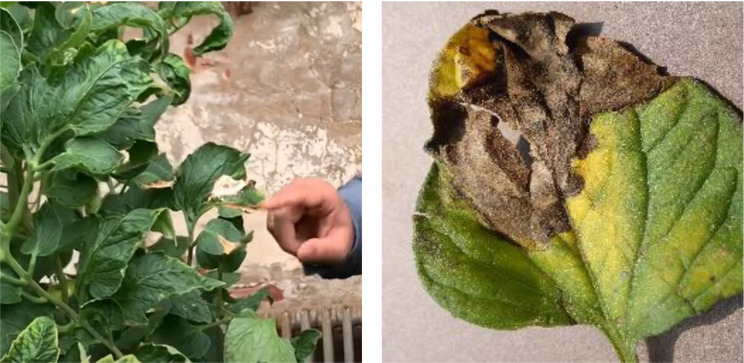
Example images for two environments. **(a)** Images captured in natural environments, **(b)** Images captured in controlled environments

Traditional methods for detecting and identifying plant diseases have shown some degree of success through manual feature extraction [12, 13]. However, such methods require a strong professional background and knowledge reserves, rendering them highly subjective. Additionally, some valuable features that cannot be discerned by the naked eye are easily overlooked. Furthermore, when faced with massive amounts of data in natural environments, the accuracy of these traditional methods is significantly reduced [14, 15]. Compared to traditional methods, deep learning has powerful feature expression capabilities and can automatically extract features from massive multi-type disease data for detection and identification, thus achieving better results [16, 17]. The performance of deep learning models in detection is inseparable from the training dataset. Currently, datasets for agricultural disease detection models are primarily categorized into two types: those captured in natural environments (with backgrounds) and those in controlled conditions (without backgrounds). As illustrated in Fig. 4, images collected in natural environments feature complex backgrounds, resulting in models with better robustness and generalization. Conversely, images captured in controlled environments lack background interference, leading to models that may not perform well in natural settings.

Given the close relationship between tomato disease occurrence and agricultural practices, management levels, and climate change, existing open datasets for tomato disease primarily consist of laboratory samples, such as AI Challenger 2018, Kaggle, PlantVillage, among others. Moreover, due to the considerable time and effort required to collect a sufficient number of samples from natural environments, research on tomato disease detection models often relies on open datasets captured in controlled conditions for training purposes.

Models trained on natural environment samples primarily focus on PC-based model construction, improvement, and structural analysis, neglecting the requirements for lightweight and high precision in practical applications. This considerable gap distances them from meeting the demands of automated disease detection in real planting scenarios. For example, Li et al. (2019) [18] developed an early detection platform for tomato late blight based on smartphones while Sun et al. (2021) [19] collected 2230 photographs depicting five prevalent apple leaf disease types with simple laboratory backgrounds and complex orchard backgrounds. Using data augmentation technology, they generated 26,767 training images and proposed a mobile-based detection model, MEAN-SSD, and the algorithm achieved an impressive detection accuracy of 83.12% while maintaining a swift processing speed. Similarly, Zhang et al. (2021) [20] introduced skip connections into Faster R-CNN to obtain an exceptional detection accuracy of 83.12% and a rapid processing speed on a self-built soybean disease image dataset. Chen et al. (2021) [21] developed and implemented a model for three types of cucumber leaves - leaf mold, bacterial angular leaf spot, and healthy. By integrating an effective backbone network, feature fusion module, and predictor, the system achieved enhanced performance through the fusion of feature maps at various levels, and the detection accuracy reached 85.52%. Fang (2021) [22] et al. proposed a novel self-supervised cross-iterative clustering approach for the analysis of unlabeled plant disease images, presenting a valuable contribution to the field of automated plant disease diagnosis and classification. Moreover, Dananjayan et al. (2022) [23] fine-tuned and evaluated multiple detectors. The results showed that YOLOv4 could achieve swift and precise disease detection capabilities. Kundu et al. (2022) [24] presented a study on disease detection and severity prediction in maize crops. Paymode and Malode (2022) [25] conducted research on multi-crop leaf disease image classification using transfer learning. Qi et al. (2022) [26] introduced an enhanced network model called SE-YOLOv5s, which added visual attention mechanisms to the YOLOv5s model to achieve key feature extraction. Experimental results on a tomato disease test set showed an accuracy of 91.07%. Syed-Ab-Rahman et al. (2022) [27] introduced an innovative approach utilizing an end-to-end anchor-based deep learning model for the detection and classification of citrus diseases, offering promising prospects for automated disease monitoring in agriculture. These studies underscore the growing importance of artificial intelligence and deep learning in agriculture, providing innovative solutions for crop health and production management. While these disease detection models have realized a real-time return of disease recognition results, it is worth noting that models developed for single plant diseases are difficult to generalize due to differences in plant biology and diseases [28].

Models developed based on ideal environment samples often lack practical validation of disease detection accuracy in natural environments. Existing research indicates that models developed based on ideal environment samples are only suitable for detecting diseases when the disease pixels dominate the image content. However, images obtained in natural settings are characterized by complex backgrounds, lighting interference, varying shooting angles, and diverse lesion scales, making it difficult for the model to be directly applied. Furthermore, the model fails to autonomously adapt to disturbances caused by changes in field lighting, leaf distortion, and variations in lesion angles and poses, resulting in poor performance in natural environments. The system proposed by Bora et al. (2023) [29] achieved disease detection rates of 99.84%, 95.2%, 96.8%, and 93.6% for tomato leaves, stems, fruits, and root positions, respectively. Zhang et al. (2023) [30] reported experimental results on 3123 tomato leaf images, including 1850 camera-captured images and 1273 obtained from the internet, indicating that the proposed M-AORANet achieved a recognition accuracy of 96.47%. Sunil et al. (2023) [31] utilized a Multi-Feature Fusion module (MFFN) to classify a publicly available tomato disease dataset, achieving training, validation, and external testing accuracies of 99.88%, 99.88%, and 99.83%, respectively. These models have demonstrated excellent disease classification results in ideal environments but only provide information on the type of disease without localizing the lesions, making it challenging to extend them to natural environments.

**Fig. 5:**
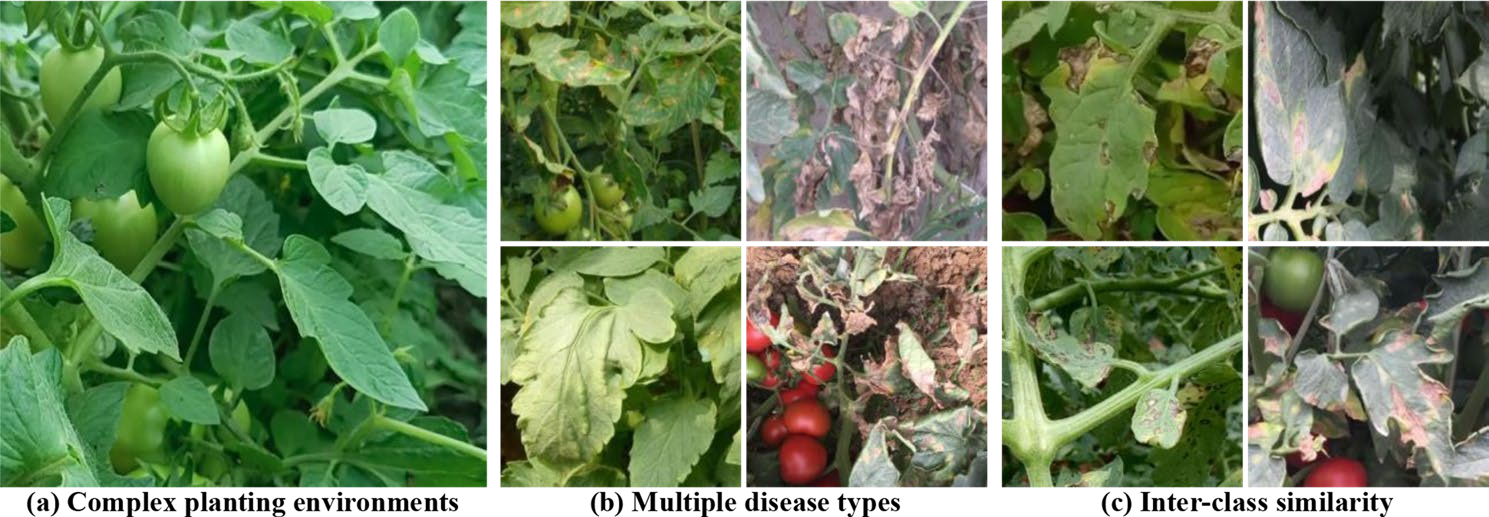
The challenges of tomato disease detection task

The detection of tomato diseases using machine vision poses significant challenges [32], such as complex planting environments, multiple disease types, and inter-class similarity. The tomato diseases detection algorithm is required to have high capabilities in multi-feature extraction and cross-scale analysis [33]. Despite recent progress in deep learning technology addressing these issues [34, 35], improving the accuracy of tomato disease detection and meeting multi-region, multi-space, and multi-time disease detection requirements in greenhouse cultivation remains an important concern. This study presents a deep-learning approach to detect tomato diseases. We analyze the types and characteristics of tomato diseases to improve and experiment with the algorithm repeatedly. Our method meets the precision and speed requirements for intelligent detection of tomato diseases, thereby reducing the cost of manual diagnosis (See Fig. 5).

Drawing on the foregoing analysis and insights from human brain neuroscience, this study reframes the task of tomato disease object detection into a reasoning challenge, focusing on determining the disease category for each object and pinpointing the disease location. To this end, we propose the fusion of the Transformer and YOLOv8n models, culminating in the TomatoDet framework, tailored to detect small and occluded objects effectively. Our specific innovations include:

1. Establishing a feature extraction module that amalgamates the self-attention mechanism of Swin-DDETR, bolstering information extraction for small-scale objects through a novel backbone feature extraction network. This approach accelerates convergence speed and enhances detection performance without augmenting model complexity.
2. Integration of the dynamic activation function Meta-ACON with the backbone network, facilitating the capture of global information and enhancing object detection performance.
3. Introduction of the proposed Bidirectional Weighted Feature Pyramid Network (IBiFPN) to fuse multi-scale features, thereby enhancing the discriminative ability of disease objects and effectively mitigating the omission and misidentification of occluded disease objects in complex backgrounds.
4. Experimental validation on a tomato disease dataset illustrates the efficacy of the proposed TomatoDet in achieving superior performance, meeting the demands for real-time detection of tomato diseases in greenhouse environments.

## Materials and methods

**Fig. 6:**
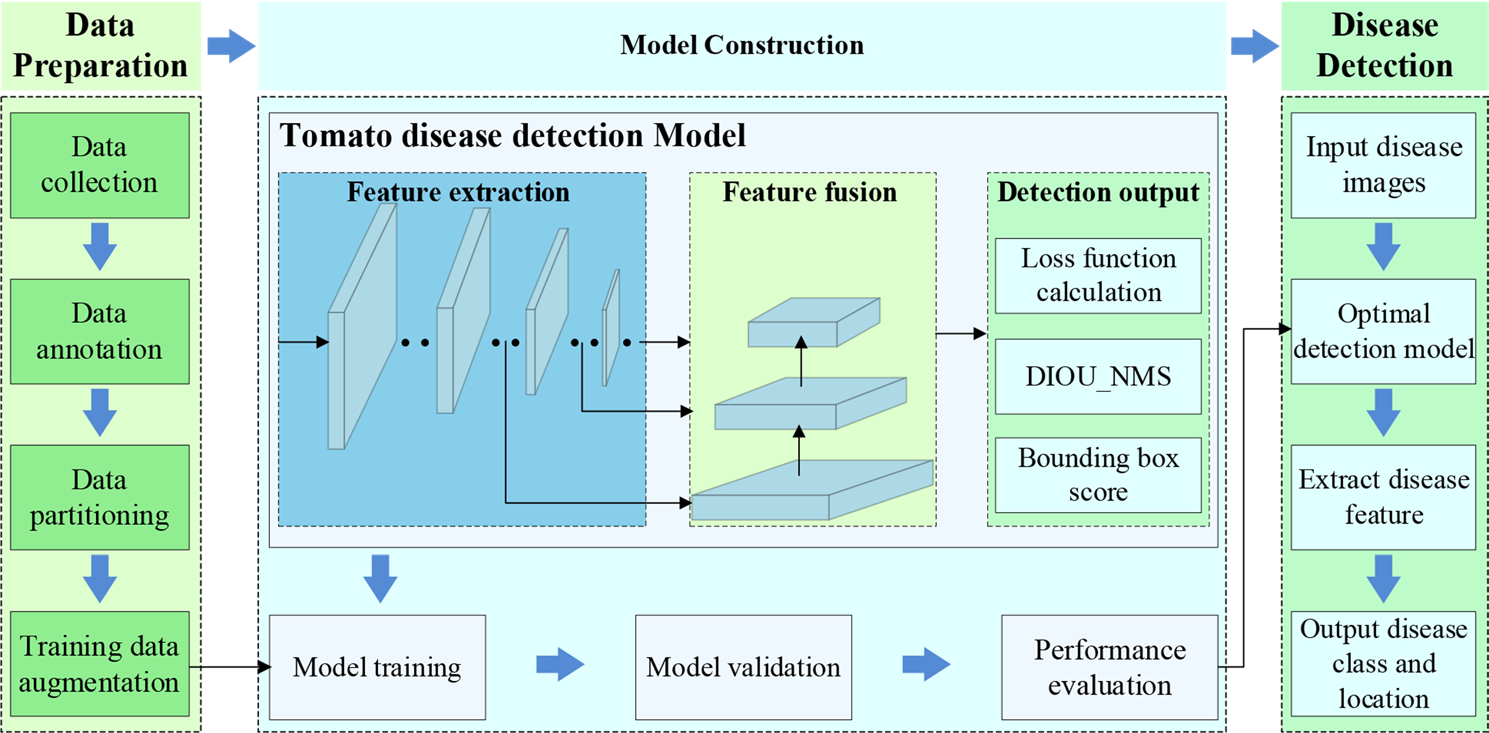
Workflow diagram of research

The research implementation diagram in Fig. 6 indicates the step-by-step accomplishment of the research work.

According to Fig. 6, the proposed tomato disease detection process in this study comprises three main parts: data preparation, construction of the tomato disease detection model, and tomato disease detection.

1. After obtaining greenhouse tomato disease images, initial screening is conducted to eliminate images of relatively low quality. This process constructs the initial set of disease images, performs data labeling, and partitions the dataset. Since training convolutional neural networks requires a large amount of training data, data augmentation methods are employed to expand the disease training set further, aiming to enhance disease recognition accuracy and prevent overfitting.
2. After establishing the dataset, tailored feature extraction and feature fusion modules are constructed based on the requirements of tomato disease detection. A tomato disease detection model is proposed and trained, validated, and evaluated.
3. The model is tested using a test dataset, and the optimal model is selected. It is then used to identify disease categories and provide location information for input disease images.

### Feature extraction module Swin-DDETR

In complex backgrounds, the background for tomato diseases is complicated, and the size of disease spots is small. As weather, lighting, and occlusion affect imaging, disease spot imaging poses diverse postures, blurry details in symptom features, high missed warnings, and false alarm rates due to overlapping occlusions. Additionally, existing large-scale servers cannot be used for tomato planting environments, making it necessary to embed the model into a mobile terminal. Thus, there are high requirements for feature extraction. As the primary neural network of the object detection model, the feature extraction part directly determines its effectiveness in identifying and classifying objects.

Motivated by the attention mechanism [36], the Transformer framework [37], the Detection Transformer framework [38] and the Deformable DETR framework [39], this study proposes a Swin-DDETR module to strengthen feature extraction.

**Fig. 7:**
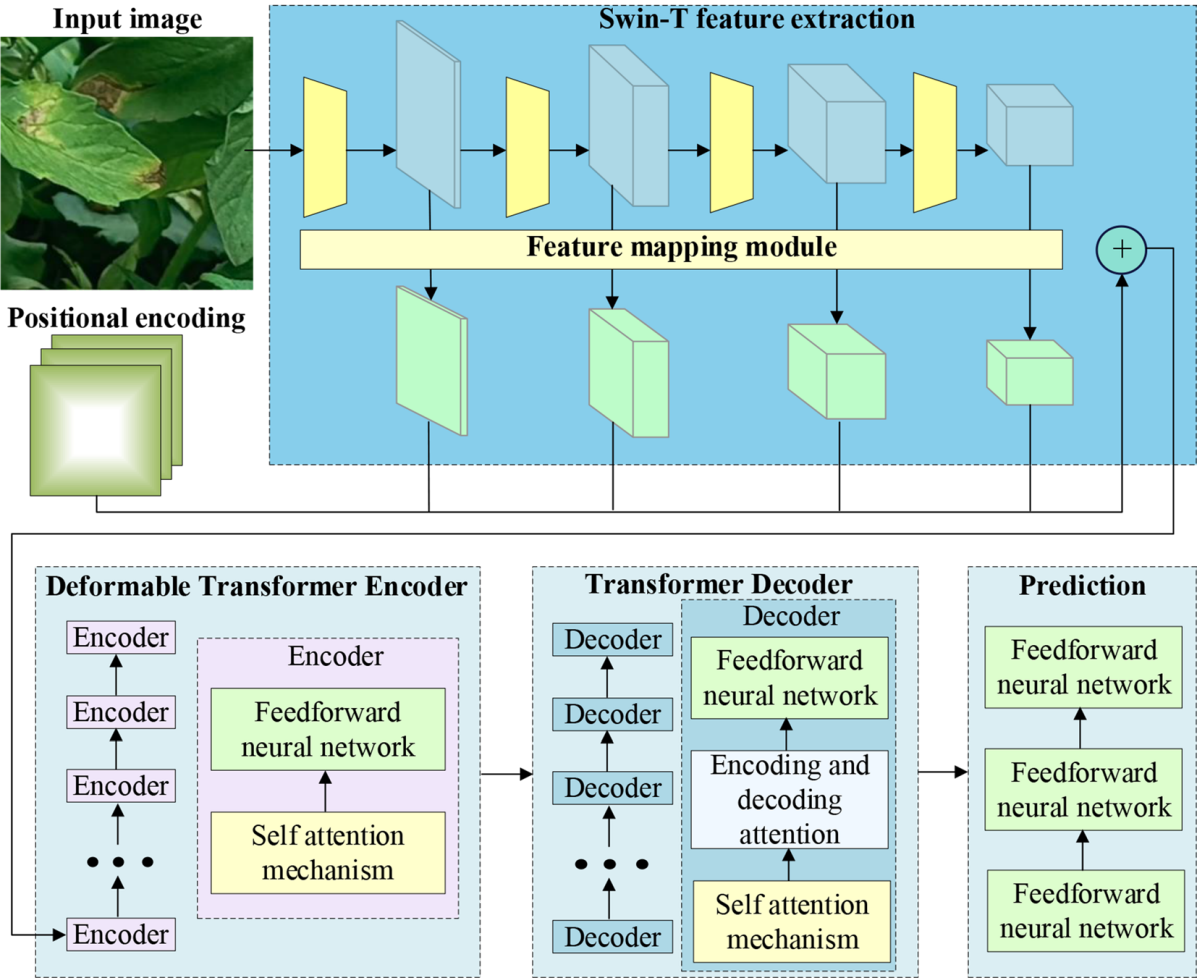
Structure of Swin-DDETR

The proposed Swin-DDETR module is shown in Fig. 7. To encode multi-scale feature maps, a deformable attention encoder is used instead of the attention encoder [40]. This allows the algorithm to naturally aggregate multi-scale features and enhance its detection ability for small objects.

**Fig. 8:**
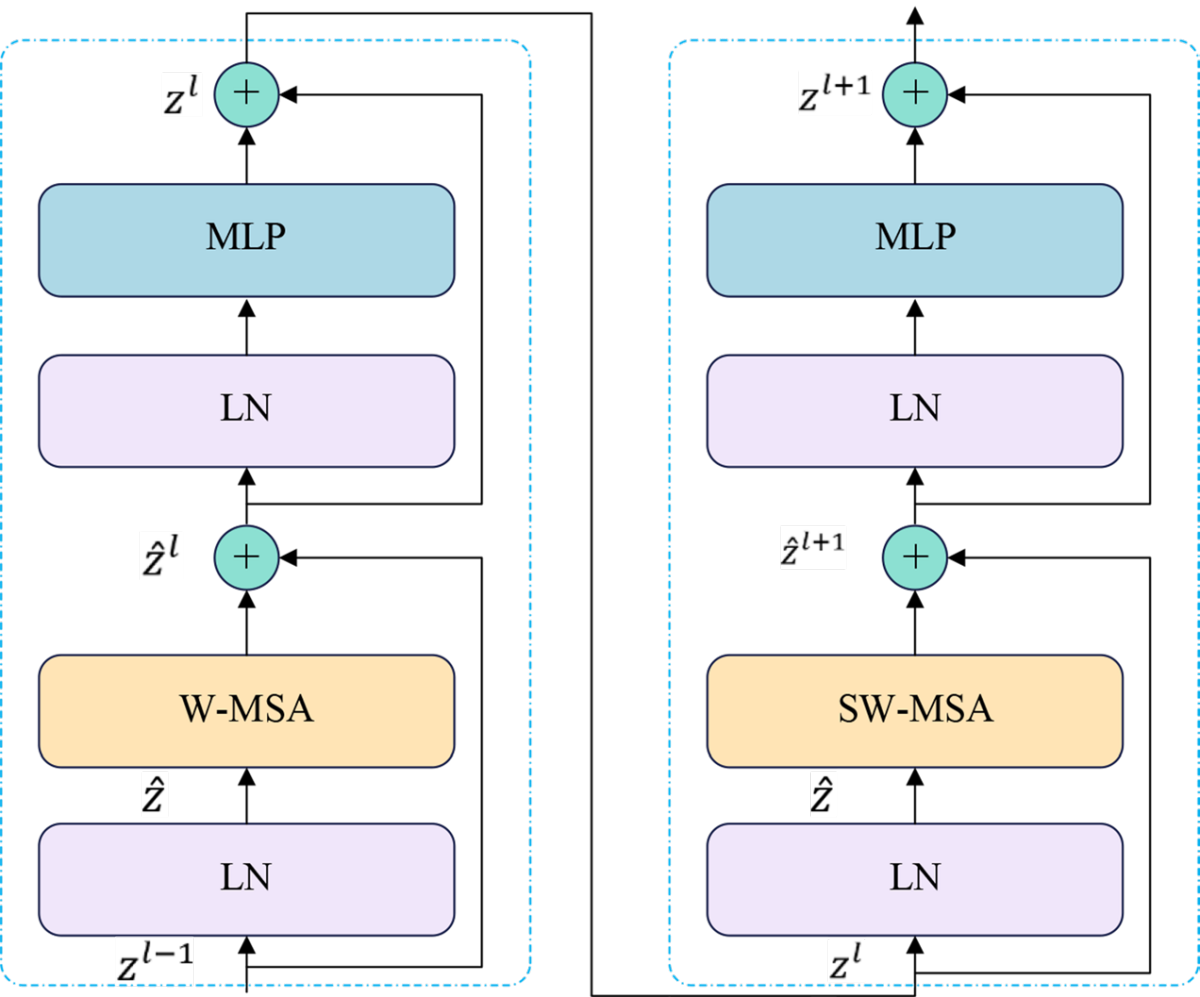
Structure of two successive Swin Transformer blocks

Swin-DDETR introduces the Swin Transformer [41], which is based on the offset window attention mechanism, to replace ResNet for modeling complex scenes and constructing feature maps with richer semantic information. The standard DDETR model uses ResNet as the feature extraction network, resulting in a smaller receptive field of the convolutional kernel compared to the Transformer. This limitation hinders the effective extraction of high-level semantic information from images and makes it challenging to reason over long distances, especially for complex scenes in tomato disease images. To mitigate the complexity of the feature extraction network, the Swin Transformer with the Swin-T structure is employed for feature extraction. Swin-T and ResNet-50 exhibit similar complexity, as depicted in the basic block structure shown in Fig. 8.

Within the Swin-T feature extraction network, the input image undergoes four stages of computation to sequentially generate feature maps with varying resolutions and channel numbers. At each stage, the features from the previous stage undergo initial block slicing and linear embedding. Subsequently, they are input into a series of stacked Swin Transformer basic blocks for processing, as computed within each Swin Transformer block using the following equation:

1 $${\widehat{z}}^{l}=W-MSA\left(LN\left({z}^{l-1}\right)\right)+{z}^{l-1}$$

2 $${z}^{l}= MLP\left(LN\left({\widehat{z}}^{l}\right)\right)+{\widehat{z}}^{l}$$

3 $${\widehat{z}}^{l+1}=SW-MSA\left(LN\left({z}^{l}\right)\right)+{z}^{l}$$

4 $${z}^{l+1}= MLP\left(LN\left({\widehat{z}}^{l+1}\right)\right)+{\widehat{z}}^{l+1}$$

In the aforementioned formula, W-MSA represents window multi-head attention, SW-MSA stands for offset window multi-head attention, ${\widehat{z}}^{l}$ and ${z}^{l}$ denote the features outputted from the $l$ offset window multihead attention module and the multilayer perceptron module, respectively. Following the feature extraction of the input image by Swin-T, a multiscale feature map with four different scales is obtained. The feature map generated in the $i$ stage is denoted as:

5 $${C}_{i}\in {R}^{\frac{H}{{2}^{i}}\times \frac{W}{{2}^{i}}\times {c}_{i}}$$

In the aforementioned formula, H and W represent the height and width of the input image, and ${c}_{i}$ denotes the number of feature map channels. The formula is as follows:

6 $${c}_{i}=3\times {2}^{i+3}$$

The standard DDETR model primarily focuses on object detection in the COCO dataset, which primarily consists of natural scenes. However, the proportion of small and medium-sized objects in COCO dataset is significantly lower than that in tomato disease images. As a result, the standard DDETR model is adversely affected by the lack of low-level features, resulting in lower accuracy in detecting small and medium-sized objects. In the COCO dataset, objects with pixel areas smaller than 32 × 32 are defined as small objects, those with areas between 32 × 32 and 96 × 96 are defined as medium-sized objects, and the rest are considered large objects.

From Table 1, it can be observed that in the tomato disease dataset, the proportion of small and large objects differs significantly from the COCO dataset, with a 23% higher proportion of small objects and a 23% lower proportion of large objects. Furthermore, over 99% of the objects in the tomato disease dataset are categorized as small or medium-sized objects, indicating a notable disparity in object distribution compared to the COCO dataset.

**Table 1:** Scale distribution of objects in the tomato disease dataset and the COCO Dataset

Dataset	Small object ratio	Medium object ratio	Large object ratio
COCO	0.42	0.34	0.24
Ours	0.65	0.34	0.01

In the Swin-DDETR feature extraction network, a feature mapping module is introduced to enhance the utilization of the ${C}_{2}$ feature map from Swin-Transformer, without downsampling the ${C}_{5}$ feature map. This improvement increases the proportion of low-level features in the constructed multiscale features, reducing the minimum downsampling rate from 8 to 4, thereby preserving more fine-grained details from the input image. In contrast, the standard DDETR model only utilizes the ${C}_{3}$, ${C}_{4}$, and ${C}_{5}$ level features from ResNet, neglecting the use of the lower-level ${C}_{2}$ feature map. This leads to a high minimum downsampling rate in the DDETR model, resulting in the loss of a significant amount of detailed features from the original image.

**Fig. 9:**
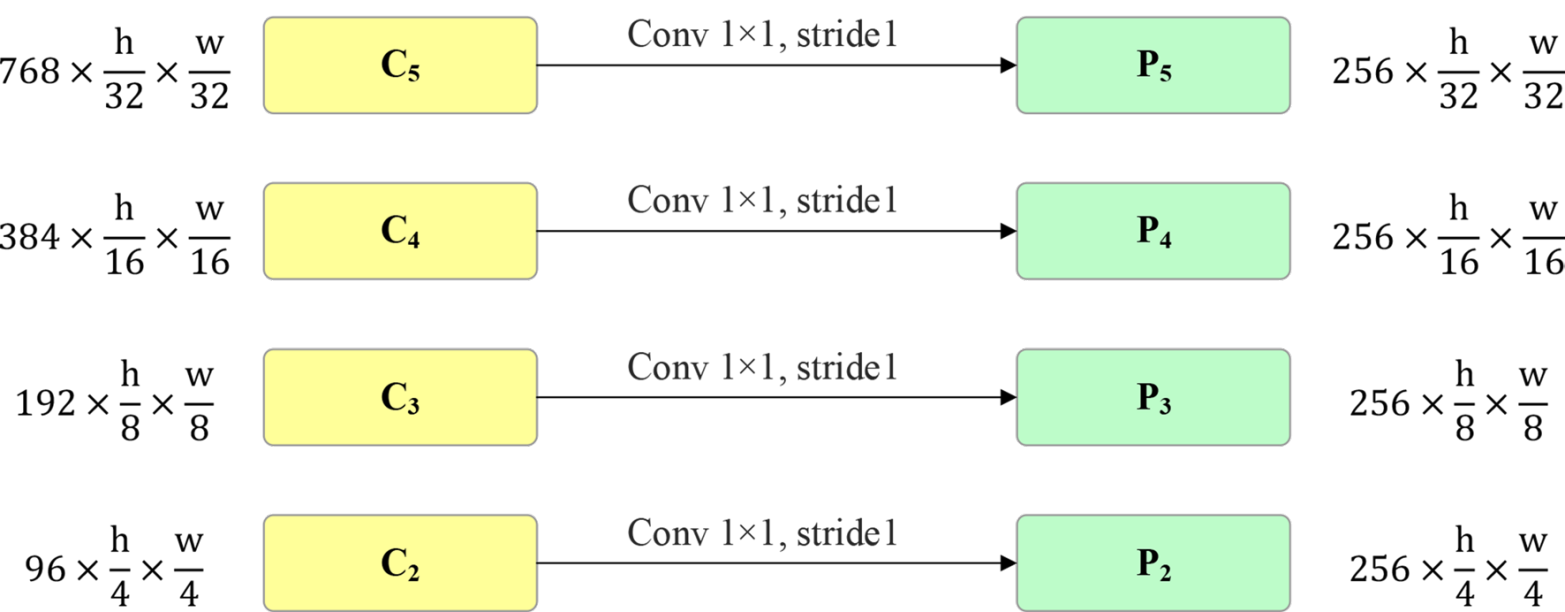
Structure of feature mapping module

The structure of the feature mapping module, as shown in Fig. 9, employs 1 × 1 convolutional operations to aggregate information from different channels. The same number of convolutional kernels is applied to feature maps $${C}_{2},$$$${C}_{3}$$, $${C}_{4}$$, and $${C}_{5}$$with varying channel numbers, resulting in multiscale features $${P}_{2},$$$${P}_{3}$$, $${P}_{4}$$, and $${P}_{5}$$ with consistent feature dimensions, maintaining uniform embedding dimensions in the Transformer.

Swin-DDETR module can supplement the global information that is lacking in convolution operation and highlight the representation of small targets on the feature map, thereby enhancing the capacity to detect and track diminutive objectives. Consequently, Swin-DDETR module was added to the final phase of the backbone network of the baseline, resulting in a new feature extraction module named Swin-DDETR specifically for tomato disease detection. This module concentrates on the relevant segments of the input for greater efficiency, weakens interference from complex backgrounds, enhances the targeted learning of disease information features, and significantly improves the efficiency of model training by outputting all predicted results at once during feature map processing, as opposed to traditional Transformers.

### Dynamic activation function Meta-ACON

The utilization of activation functions can enhance the network’s ability to learn complex mappings from data. The Mish activation function which is used in the YOLOv8n algorithm possesses characteristics such as smoothness, non-monotonicity, a boundless bottom, and a bounded top, resulting in superior performance compared to commonly used ReLU and its variants. However, it remains a static activation function that is incapable of adjusting its processing abilities for complex data by responding to different input features. To address this issue, this study introduces a dynamic activation function named Meta-ACON [42]. This allows the network to autonomously grasp the structure of the input during the learning process and determine whether neurons should be activated.

Meta-ACON can efficiently adapt to various types of data inputs with distinct patterns by automatically adjusting different parameters and selectively focusing on significant information while maintaining high accuracy. One of the most significant advantages of meta-ACON is its unique ability to facilitate robust feature extraction that lessens interference from irrelevant background information while isolating disease-related features.

In contrast to traditional activation functions, wherein an identical function applies to all input regions, Meta-ACON determines the appropriate activation function and corresponding parameter adjustments according to input characterizations, generating distinct outputs for every input of the data. Consequently, this innovative activation function enhances the precision in detecting diseases affecting tomato plants under different scenarios and addresses the challenge of detecting anomalies among complex backgrounds.

Meta-ACON is a member of the ACON (ActivateOrNot) function family. The author unified the Swish function into the ReLU function family and expanded the Maxout series of activation functions to create the ACON series of activation functions. Among them, ACON-C can be expressed as follows:

7 $$\eqalign{& {f_{ACON - C}}\left( x \right) = {S_\beta }\left( {{p_1}x,{p_2}x} \right) \cr & = \left( {{p_1} - {p_2}} \right)x \cdot \sigma \left[ {\beta \left( {{p_1} - {p_2}} \right)x} \right] + {p_2}x \cr}$$

It covers most of the current activation functions, including even more complex variations. Two learnable parameters, denoted as ${p}_{1}$ and ${p}_{2}$, enable the neural network to adaptively adjust the activation function’s shape by learning their values. A smoothing factor called $\beta$ is employed to control whether a neuron should be activated or not. In ACON-C, $\beta$ is set as a hyperparameter and requires manual tuning. Meta-ACON enhances upon ACON-C and introduces an adaptive function to compute the smoothing factor $\beta$ automatically. This enhancement facilitates dynamic control of the neurons’ activation status based on the input feature matrix $x$.

The adaptive function is designed to target the channel space, as shown in the following formula:

8 $${\beta }_{c}=\sigma {W}_{1}{W}_{2}\sum _{h=1}^{H}\sum _{w=1}^{W}{x}_{c,h,w}$$

Initially, the mean for dimensions H and W is calculated. This is followed by adjusting the number of channels using two 1 × 1 convolutional layers. Finally, the Sigmoid activation function is employed to confine the ultimate output of ${\beta }_{c}$ within the range of (0, 1), thereby controlling whether the neuron is activated. ${\beta }_{c}$ represents the shared parameter along the channel dimension, while W1 and W2 are the parameters of the two convolutional layers. The formula is as follows:

9 $${W}_{1}\in {R}^{C\times C/r}$$

10 $${W}_{2}\in {R}^{C/r\times C}$$

In the above formula, C denotes the count of channels, whereas r signifies the scaling factor between the two convolutional layers, which has been set at 16 to optimize parameter usage. This study introduces a new activation layer formed through the replacement of the activation function with Meta-ACON. The new activation layer has replaced all activation layers in the main network Swin-DDETR. The use of Meta-ACON empowers this innovative architecture to make corresponding transformations based on different inputs and adaptively determine its degree of nonlinearity – enabling the network to better fit various data distributions. This feature provides superior performance when dealing with small object detection, particularly with many samples and complex distribution. With this new approach, the network can classify positive and negative samples more efficiently and improve the overall generalization performance.

### Feature fusion module IBiFPN

As compared to the entire tomato plant in a complex environment, disease spots belong to smaller targets that are easily affected by background interference. Feature fusion combines the feature with the rich semantic information derived from deep feature maps to enhance the capability of detecting small targets. The objective of this research is to enhance the model’s capacity to detect tomato disease targets and effectively fuse feature information at varying scales. To achieve this objective, we construct a feature fusion module following the feature extraction module. This module incorporates concat layers, convolutional layers, and C2f modules to execute an additional upsampling and downsampling process.To achieve feature fusion, the Concat layer is utilized to integrate the feature layer of identical scale from the backbone network, which captures a greater amount of detailed feature information for smaller objects and enhances the sensitivity of the new detection layer to small target features.

The BiFPN structure [43] is mainly used to fully fuse feature maps with different resolutions. Compared with FPN (Feature Pyramid Networks) in common target detection algorithms, BiFPN improves the feature fusion performance in the following aspects: using jump connections to lighten the network; adding an attention mechanism to weight the learning of more critical feature information; and setting up two paths of up-sampling and down-sampling for more complete feature fusion.

**Fig. 10:**
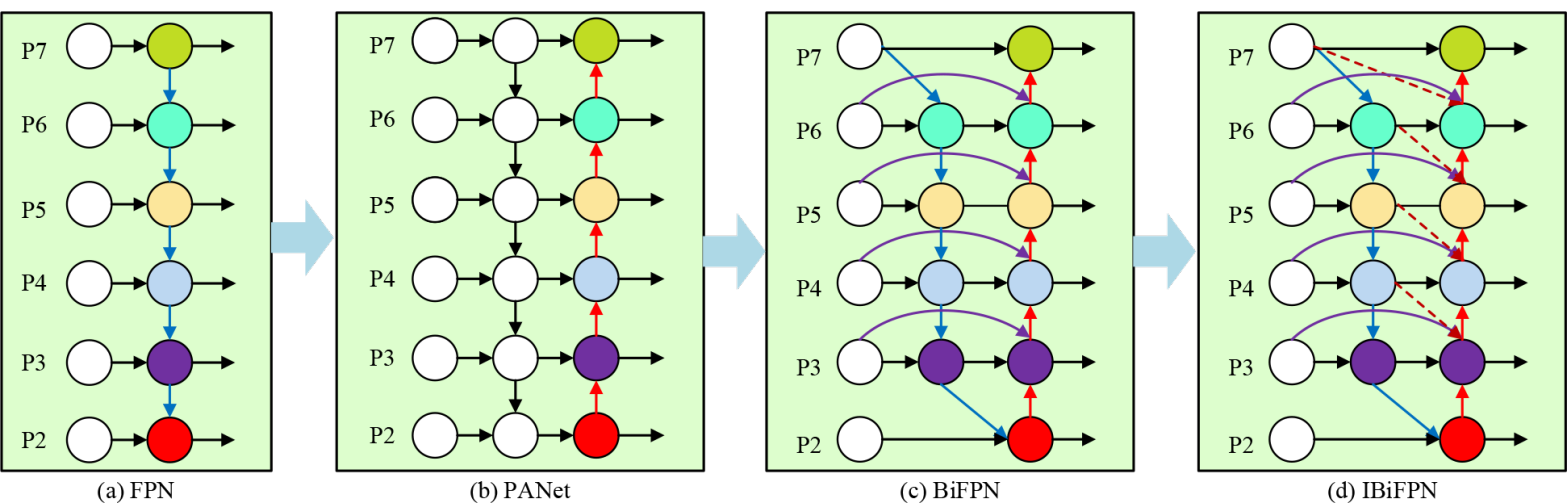
Comparison of four feature fusion structures

To enhance the small target detection capability of BiFPN, this study introduces an enhanced version termed Improved BiFPN (IBiFPN) (Fig. 10d), which is contrasted with FPN (Fig. 10a), PANet (Fig. 10b), and the conventional BiFPN structure (Fig. 10c). The specific fusion path of the proposed IBiFPN structure (Fig. 9d) is as follows: intermediate information is obtained by up-sampling, taking the intermediate point P5-td as an example: its added attention mechanism fuses the up-sampling information of P6-td and the input information of P5 itself; output information is obtained by down-sampling, taking the output point P4-out as an example: its added attention mechanism fuses the down-sampling information of P3-out, the intermediate information of P4-td, the small-scale information of P5-td, and the input information of P4 itself; finally, P3-out, P4-out, P5-out, P6-out, and P7-out are obtained by analogy and passed to the next layer of the proposed IBiFPN feature fusion structure as input information.

In this study, fast regularization methods are utilized to weight the input feature maps of nodes. The weighted formula for each feature fusion node is as follows:

11 $$O=\sum _{i}\frac{{w}_{i}}{\epsilon +\sum _{j}{w}_{j}}\cdot {I}_{i}$$

In the formula, ${w}_{i}\ge 0$epsilon is a small value set to 1 × 10^−4^ for stability calculation. It can be observed that the weight range of feature fusion is between 0 and 1, which avoids the use of the softmax function which leads to significant increases in computation time. For instance, in the 4th layer, the BiFPN structure performs cross-scale connections and weighted feature fusion using the following process:

12 $${P}_{4}^{td}=Conv\left(\frac{{w}_{1}\cdot {P}_{4}^{in}+{w}_{2}\cdot Resize\left({P}_{5}^{in}\right)}{{w}_{1}-{w}_{2}+\epsilon }\right)$$

13 $${P}_{4}^{out}=Conv\left(\frac{{w}_{1}^{{\prime }}\cdot {P}_{4}^{in}+{w}_{2}^{{\prime }}\cdot {P}_{4}^{td}+{w}_{3}^{{\prime }}\cdot Resize\left({P}_{3}^{out}\right)}{{w}_{1}^{{\prime }}+{w}_{2}^{{\prime }}+{w}_{3}^{{\prime }}+\epsilon }\right)$$

Here, ${P}^{in}$ denotes the input features, ${P}_{out}$ represents the output features, and ${P}^{td}$ denotes the intermediate layers in the top-down feature fusion process.

**Fig. 11:**
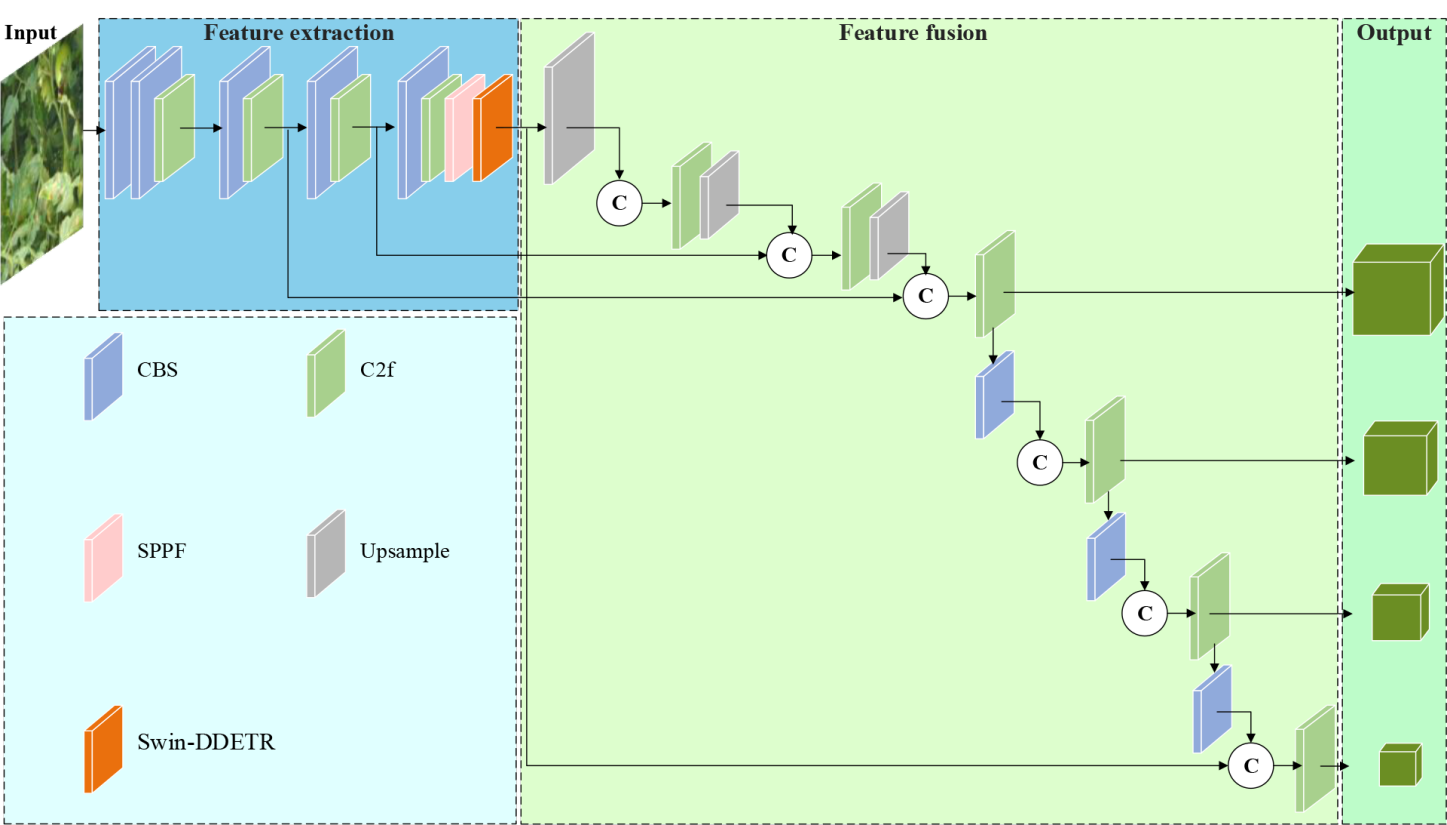
General framework of tomato disease detection model (TomatoDet)

The proposed feature fusion module is capable of acquiring global information and exhibits strong feature fusion and fitting ability. Consequently, the model’s ability for detecting tomato diseases is enhanced. Therefore, as illustrated in Fig. 11, we present an overall framework of our tomato disease detection model (TomatoDet).

### Data collection

**Fig. 12:**
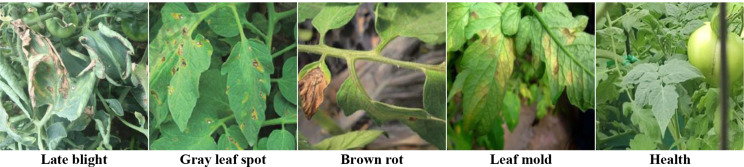
Sample of the tomato disease dataset

For the experiment, we employed a tomato disease dataset that we created from scratch. The images were captured using an agricultural Internet of Things monitoring device (HS-CQAI-1080) from a tomato cultivation facility situated in Shouguang City, located in Shandong Province, China. (Longitude coordinates: 118.782956 E, latitude coordinates: 36.930686 N). The pixel dimension for the captured images is 3648 × 2056. The capture period for the images spanned from January 1st to December 31st of 2022 and occurred during two distinct periods, namely from 08:30 − 11:30 and 14:30 − 17:30. During image capture, the equipment was positioned at a distance between 0.2 and 0.5 m from the diseased leaves. The experimental dataset comprises over 10,000 natural environmental images, devoid of structured backgrounds, collected from various conditions including sunny and cloudy weather, with different disease locations and states. The dataset includes four common tomato diseases and healthy leaves: late blight, gray leaf spot, brown rot, and leaf mold. Furthermore, the collected images record multiple sources of information such as the environmental temperature, location, and time of capture. The background of the disease images contains several different noises and environmental factors such as leaves, weeds, soil, and varying lighting conditions from different angles, which include backlit and front-lit. The dataset is therefore suitable for practical applications of the model. Illustrative samples of the tomato disease dataset appear in Fig. 12.

### Data annotation

**Fig. 13:**
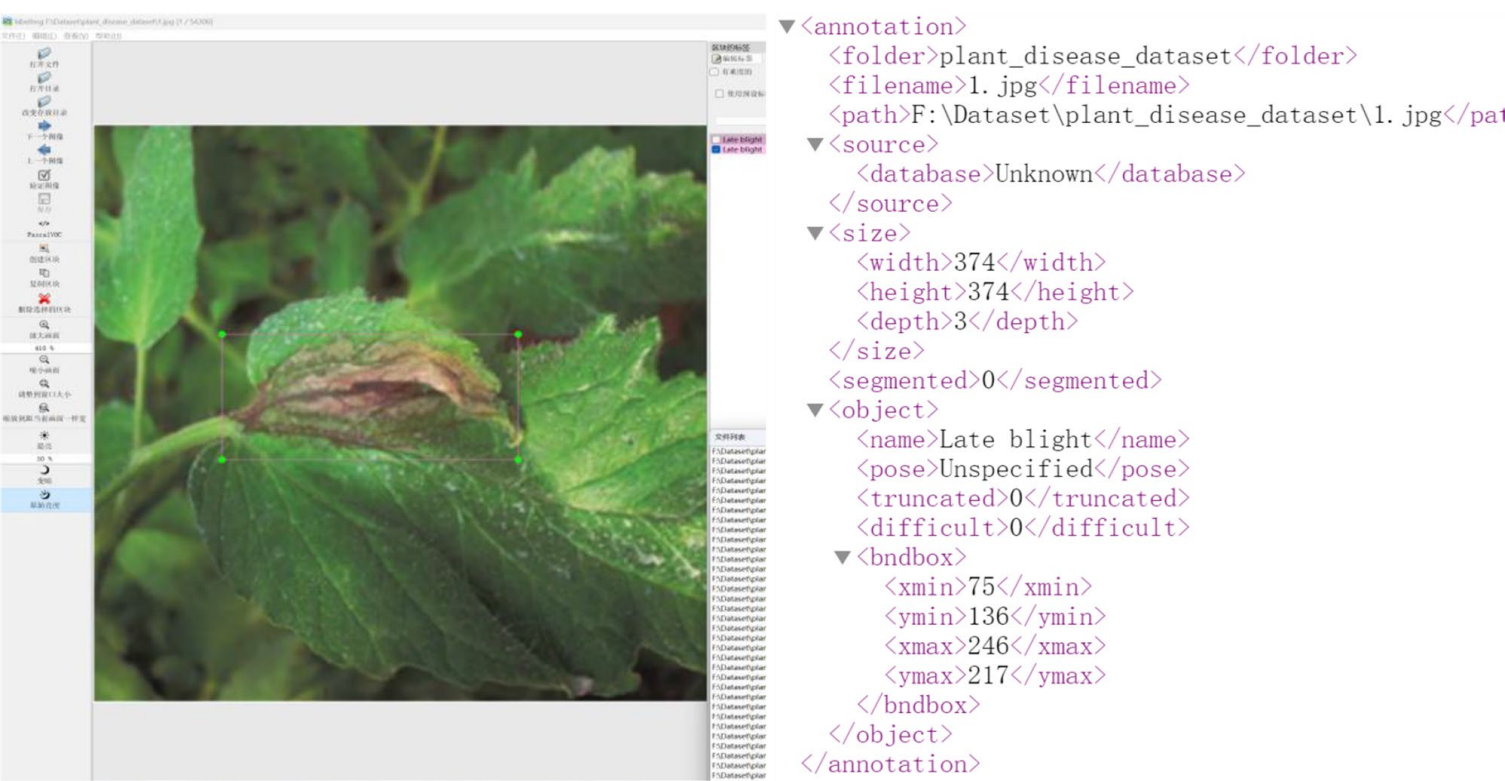
LabelImg annotation and VOC dataset format. **(a)** LabelImg annotation, **(b)** VOC dataset format

During the dataset creation process, we utilized the open-source annotation tool LabelImg to annotate the positions and categories of lesions in tomato disease images. The annotation format followed the Pascal VOC standard. Upon completion of the labeling process, an XML file was generated containing information about the image dimensions, the category of the target lesions, and the coordinates of the top-left and bottom-right corners of the lesions. The data annotation process is illustrated in Fig. 13.

### Data partitioning

From the collected data, 400 sample images were selected for each category, resulting in a total of 2,000 images. To ensure smooth experiment execution, the initial dataset was partitioned into three groups: the training set, validation set, and test set. The training set is composed of 1,600 images, while both the validation and test sets consist of 200 images each.

### Data augmentation

**Fig. 14:**
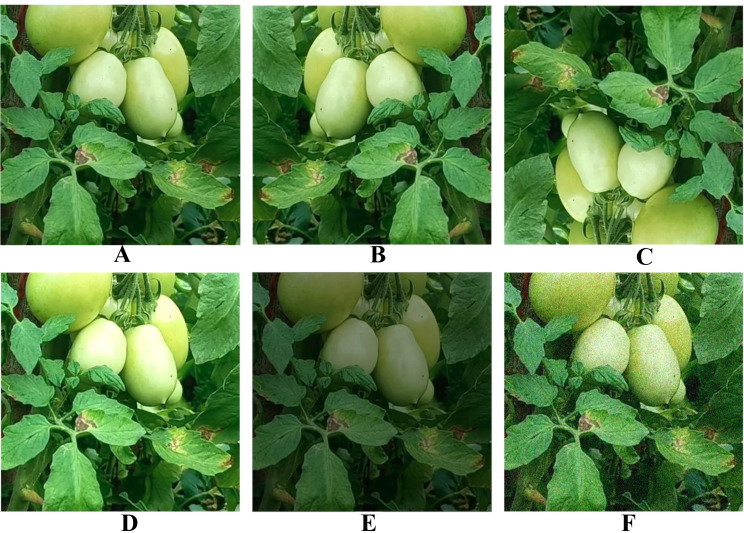
Data Augmentation of tomato images: **(A)** Original image, **(B)** Horizontal flip, **(C)** Vertical flip, **(D)** High brightness, **(E)** Low brightness, **(F)** Gaussian noise

In this study, Data augmentation methods are applied to improve the model’s ability to generalize, mitigate overfitting, and improve training effectiveness. Specifically, data augmentation was applied exclusively to the training set, while the validation and test sets were kept unaltered to accurately assess the performance. We found that this approach consistently improved the performance and generalization capability of deep learning models. Various data augmentation techniques were utilized, such as flipping the images horizontally and vertically, adjustments in brightness levels and adding Gaussian Noise as depicted in Fig. 14.

By data augmentation, the training set and validation set are kept isolated from the test set. The training set is expanded to 9600 images while the validation set and test set remain unchanged. Then the training process begins.

After data augmentation, The total count of acquired images is presented in Table 2

**Table 2:** The total count of acquired images

	Original image	Horizontal flip	Vertical flip	High brightness	Low brightness	Gaussian noise	Total
Count	1600	1600	1600	1600	1600	1600	9600

## Results

### Experimental settings

The experiments were performed in a deep learning environment on an Ubuntu 20.04 operating system orchestrated on a CUDA 11.4 architecture integrated with Pytorch 1.8.1 and MMDetection framework 2.25.1. The models were trained using NVIDIA RTX2080Ti GPU for acceleration.

Before training, the sample data were divided into multiple batches (Batch), taking into account the number of samples and the hardware environment of the computer, the Batch size was set to 32 and the number of model iterations (Epoch) was set to 100 times during the experiment in this study.

### Evaluating indicator

This study utilizes average precision mean (mAP), parameter amount (Millions) and Frames per second (FPS) to assess the performance of network models. The specific calculation formula is as follows:

14 $$Precision=\frac{TP}{TP+FP}\cdot 100\%$$

15 $$Recall=\frac{TP}{TP+FN}\cdot 100\%$$

16 $$AP={\int }_{0}^{1}p\left(r\right)dr$$

17 $$mAP=\frac{\sum _{i=1}^{K}A{P}_{i}}{K}$$

In the context of detection, true positive (TP) detections refer to correct positive predictions, false positive (FP) detections indicate incorrect positive predictions, false negative (FN) detections represent missed positive predictions, and Average Precision (AP) measures the precision of each category’s prediction, while $p\left(r\right)$ represents the Precision-Recall curve. mAP (mean Average Precision) is the average of the AP values across all categories, K denotes the number of categories being detected, while $i$ represents the current data category. The mAP is evaluated at an IoU (Intersection over Union) value of 0.5, in line with standard practice for object detection evaluation metrics. The parameter amount (Millions) represents the spatial complexity. Frames per second (FPS) represents the detection speed obtained when testing on the validation set using a Tesla T4 16GB with a batch size set to 1. The calculation formula is as follows:

18 $$FPS=\frac{{C}_{img}}{{Time}_{detect}}$$

In the above-mentioned formular, ${C}_{img}$ represents the count of images within the test dataset, and ${Time}_{detect}$ represents the time taken to detect ${C}_{img}$ images.

### Learning rate settings

**Fig. 15:**
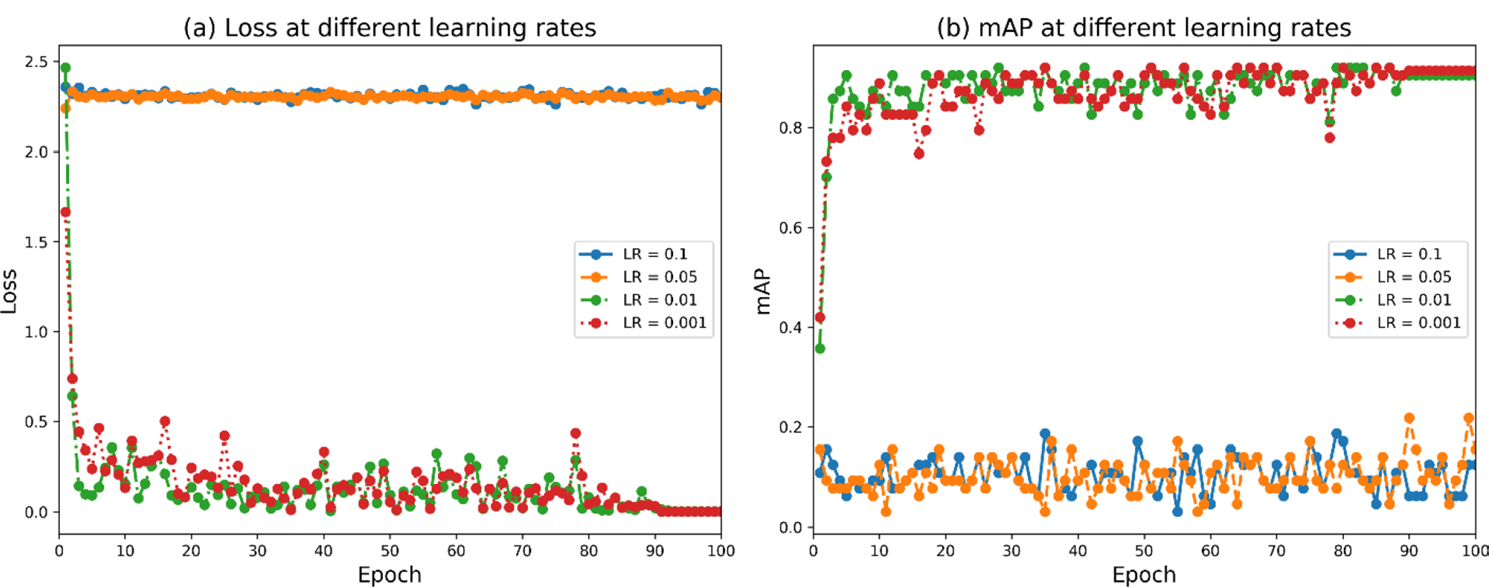
Loss function and mAP of the TomatoDet model at different learning rates

The learning rate affects the performance of the model, so here the parameter optimization is sought for different learning rates, and the experiment compares the performance of the model at learning rates of 0.1, 0.05, 0.01, and 0.001 under the same control of other conditions so that the model obtains better learning of the training set and recognition of the test set. The variation curves of the loss function for different learning rates are shown in Fig. 15a. It can be seen that the loss function is almost constant when the learning rate is 0.1 and 0.05. This indicates that the learning rate is too high and the step size is too large, which makes the function unable to achieve convergence, indicating that a moderate learning rate should be set to consider the convergence and convergence speed, comparing the convergence and convergence speed when the learning rate is 0.01 and 0.001, it can be seen that the latter is better in terms of convergence and convergence speed. In addition, from Fig. 15b, it can be seen that the accuracy fluctuates significantly in the cases of 0.1 and 0.05 learning rate, and the model accuracy is highest when the learning rate is 0.001. Therefore, the learning rate is finally set to 0.001.

### Comparison with baseline model

**Fig. 16:**
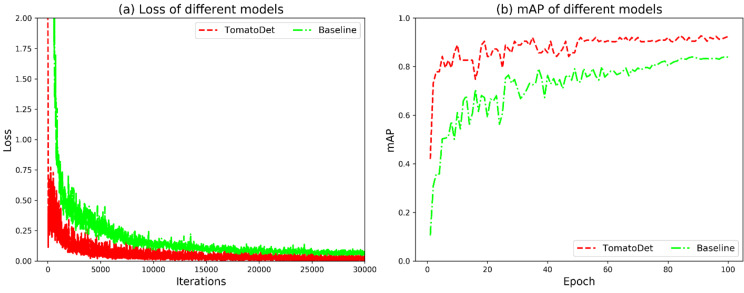
Loss function and mAP of TomatoDet and the baseline model

The proposed model and baseline model were both trained with the tomato disease image dataset constructed in this study, and the loss function and accuracy of the model after 100 epochs were obtained, as shown in Fig. 16.

As can be seen in Fig. 16a, the loss value of the proposed TomatoDet model stabilizes at about 8,000 iterations and tends to be about 0.00158, while the baseline model tends to be about 0.09969. Figure 16b shows that the proposed TomatoDet model has increased the mAP value to about 0.9 at about 55 epochs and finally stabilized at about 0.92, while the baseline model has increased the mAP value to about 0.8 at about 80 epochs and finally stabilized at about 0.83. Thus, compared to the baseline model, the proposed TomatoDet model shows a significant improvement in both loss function convergence speed and detection accuracy.

**Fig. 17:**
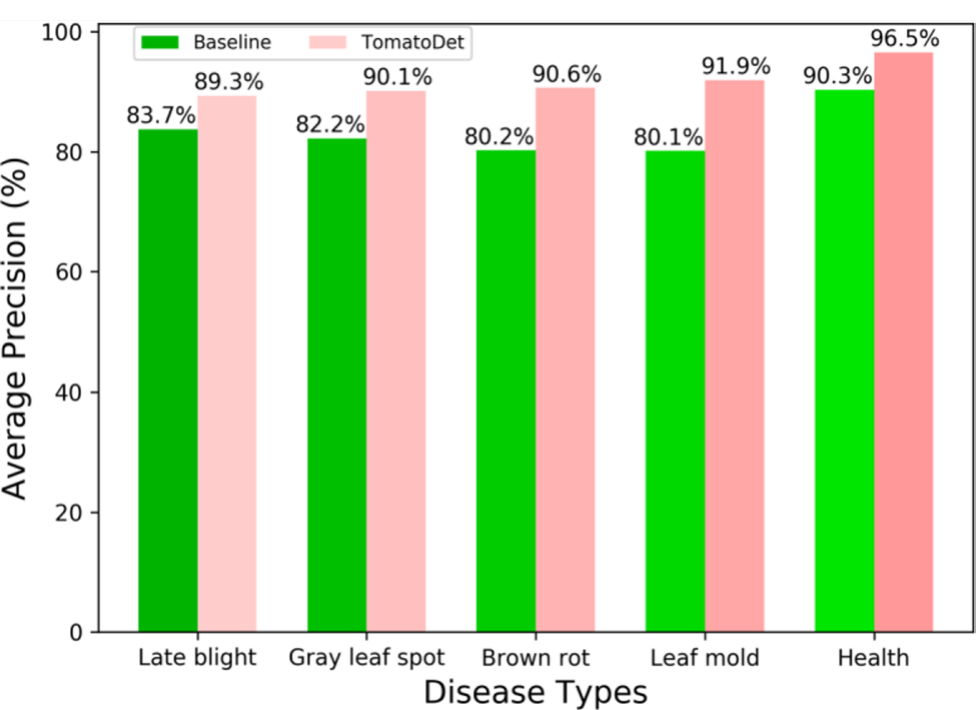
Comparison of detection effects between TomatoDet and the baseline model

Figure 17 shows that the proposed TomatoDet proposed in this study increased detection accuracy for various types of plant diseases, particularly for small targets within disease categories. This increase was especially significant for diseases such as late blight, gray leaf spot, brown rot, and leaf mold, with improvements in detection accuracy for leaf mold and similar diseases reaching 11.8% points in terms of AP value. The enhanced capability of the network in detecting small targets can be attributed to the implementation of the BiFPN feature extraction structure and the well-designed backbone network, as indicated by these findings.

### Ablation experiment

To verify if the proposed modules boost the network’s overall efficiency and determine whether there are any effects between modules, ablation experiments were employed for validation. Table 3 displays the outcomes obtained from the experiments conducted. In the table, Swin-DDETR refers to replacing the backbone network with Swin-DDETR; Meta-ACON serves as a substitution for the activation function within the backbone network; and IBiFPN indicates using the IBiFPN structure to substitute the PANet structure in the baseline.

**Table 3:** Ablation experiment results

Model	Swin-DDETR	Meta-ACON	IBiFPN	mAP (%)	FPS
1	No	No	No	83.6	42.8
2	Yes	No	No	83.2	46.5
3	No	Yes	No	82.9	34.4
4	No	No	Yes	82.8	47.8
5	Yes	Yes	No	84.3	33.9
6	Yes	No	Yes	83.7	46.9
7	No	Yes	Yes	83.6	49.8
TomatoDet	Yes	Yes	Yes	92.3	46.6

According to the results of the ablation experiment, Model 2–4 revealed that the Swin-DDETR module designed in this study has the most significant impact on network mAP, increasing it by 2.9% points compared to the original model. The introduction of the activation function Meta-ACON and the IBiFPN structure also improved the network mAP by 2.5 and 1.4% points, respectively. These results indicate that each proposed module can contribute to enhancing network detection accuracy compared to the original model and improve its ability to extract information related to tomato diseases under greenhouse environmet.

To further illustrate the influence of the proposed Swin-DDETR module on network attention, the study introduced a visual attention technique known as GradCAM [44]. This approach generates heatmaps during the network validation phase, enabling an analysis of whether the model is efficiently acquiring precise feature information by examining highlighted regions in the heat map.

**Fig. 18:**
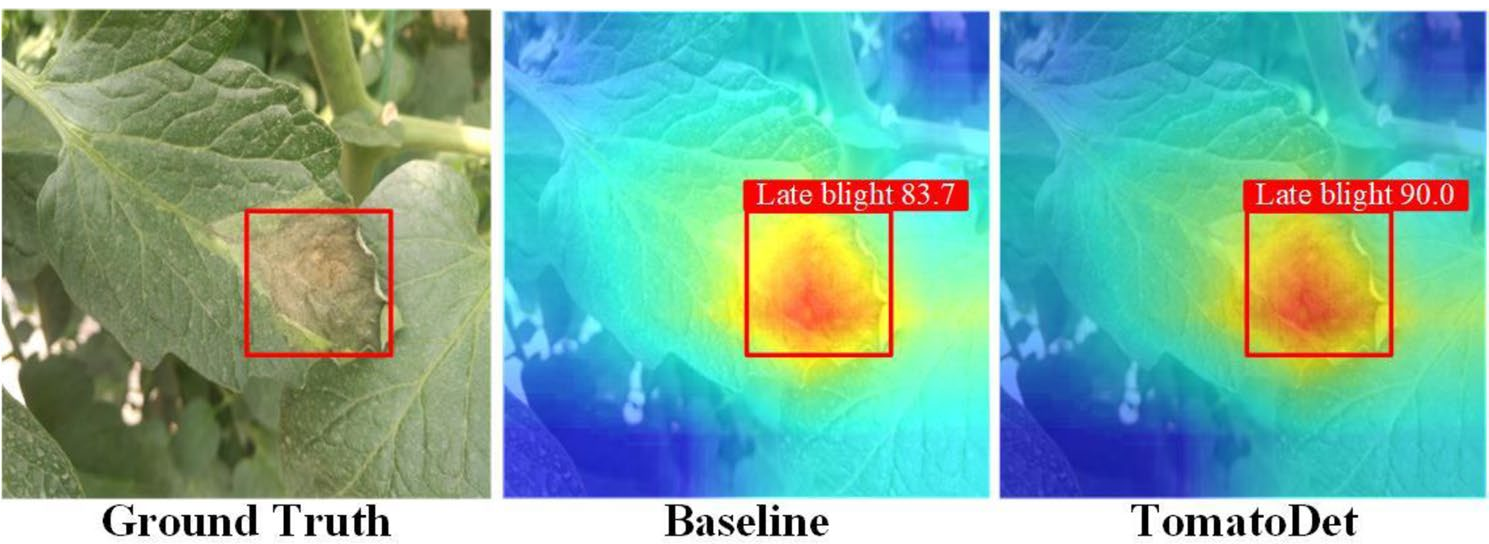
Comparison of heatmaps of baseline and TomatoDet

In Fig. 18, the attention heatmap is displayed using the GradCAM method on the validated results of disease images. The Ground Truth represents the accurate label for the disease image, the baseline refers to the original YOLOv8n model, and TomatoDet represents the proposed model. By analyzing the images, it is evident that due to the Swin-DDETR attention module’s focus on global information, the proposed TomatoDet has more attention concentrated on the disease regions than the baseline model. This feature enhances its suitability for disease detection tasks.

Experimental results from Model 5–7 evidence that the various improvement points of this study can be well incorporated. A comparison between Model 6 and Model 2 confirms that the IBiFPN structure, coupled with the new backbone network, accelerates the detection speed while simultaneously improving accuracy. These findings suggest that the IBiFPN structure can effectively enhance the feature extraction capability and reduce the complexity of the model. Ultimately, by combining all the experimental improvements, there was a mAP increase of 5.3% while adhering to real-time detection standards regarding speed.

### Comparative experiments of different algorithms

To validate the effectiveness and superiority of TomatoDet proposed in this study, we conducted comparison experiments using the TomatoDet model. We compared it with several current mainstream object detection models, including Faster R-CNN, YOLOXs, YOLOv5s, YOLOv7-tiny, and YOLOv8n. Each comparison model was trained using the same parameters and the tomato disease dataset constructed in this study. The results of these comparison experiments are presented in Table 4.

**Table 4:** Comparison results of different models

Model	mAP (%)	Parameters (Millions)	FPS	FLOPs(G)	Memory(MB)
Faster R-CNN	79.3	22.6	6.2	20.69	407.2
YOLOXs	79.8	3.4	29.2	30.98	119.6
YOLOv5s	80.9	20.6	29.8	33.05	87.9
YOLOv7-tiny	81.7	18.7	21.7	39.86	82.5
YOLOv8n	83.6	7.9	42.8	44.63	69.7
TomatoDet	92.3	13.3	46.6	48.98	43.9

The comparative experimental results demonstrate that the TomatoDet model proposed in this study outperforms Faster R-CNN, YOLOXs, YOLOv5s, YOLOv7-tiny, and YOLOv8n models in terms of both detection accuracy and speed. Moreover, it excels at detecting various categories of disease targets. These findings confirm the excellent performance of the TomatoDet model, which can efficiently and accurately identify and locate tomato disease targets even in complex backgrounds. It fulfills the deployment requirements for real agricultural scenarios.

Regarding model complexity, while the TomatoDet model slightly lags behind the YOLOX network model in terms of the number of parameters, it boasts significantly smaller memory usage than other models. This demonstrates that the Swin-DDETR structure within the TomatoDet model accelerates inference speed while efficiently reducing memory consumption. Consequently, the model is better suited for deployment on edge-end devices with limited computational power, aligning with the needs of intelligent plant disease detection development.

The AP values of the proposed TomatoDet model and other models for the detection of five categories of tomato diseases are shown in Table 5.

**Table 5:** Comparison of AP values for five types of tomato disease detection

Model	AP (%)				
	Late blight	Gray leaf spot	Brown rot	Leaf mold	Health
Faster R-CNN	80.3	78.4	77.3	78.6	88.7
YOLOXs	82.5	73.5	72.6	73.8	89.6
YOLOv5s	89.1	88.9	87.9	82.7	93.2
YOLOv7-tiny	80.4	80.4	78.1	77.6	90.1
YOLOv8n	83.7	82.2	80.2	80.1	90.3
TomatoDet	89.3	90.1	90.6	91.9	96.5

It can be seen that the AP values for various algorithms differ significantly in the detection of different disease categories, with only minor distinctions in detecting healthy tomatoes. The TomatoDet model proposed in this study exhibits distinct advantages in detecting various disease categories. This reaffirms the model’s exceptional capability to detect multi-scale and multi-category tomato disease objects.

### Model detection performance

**Fig. 19:**
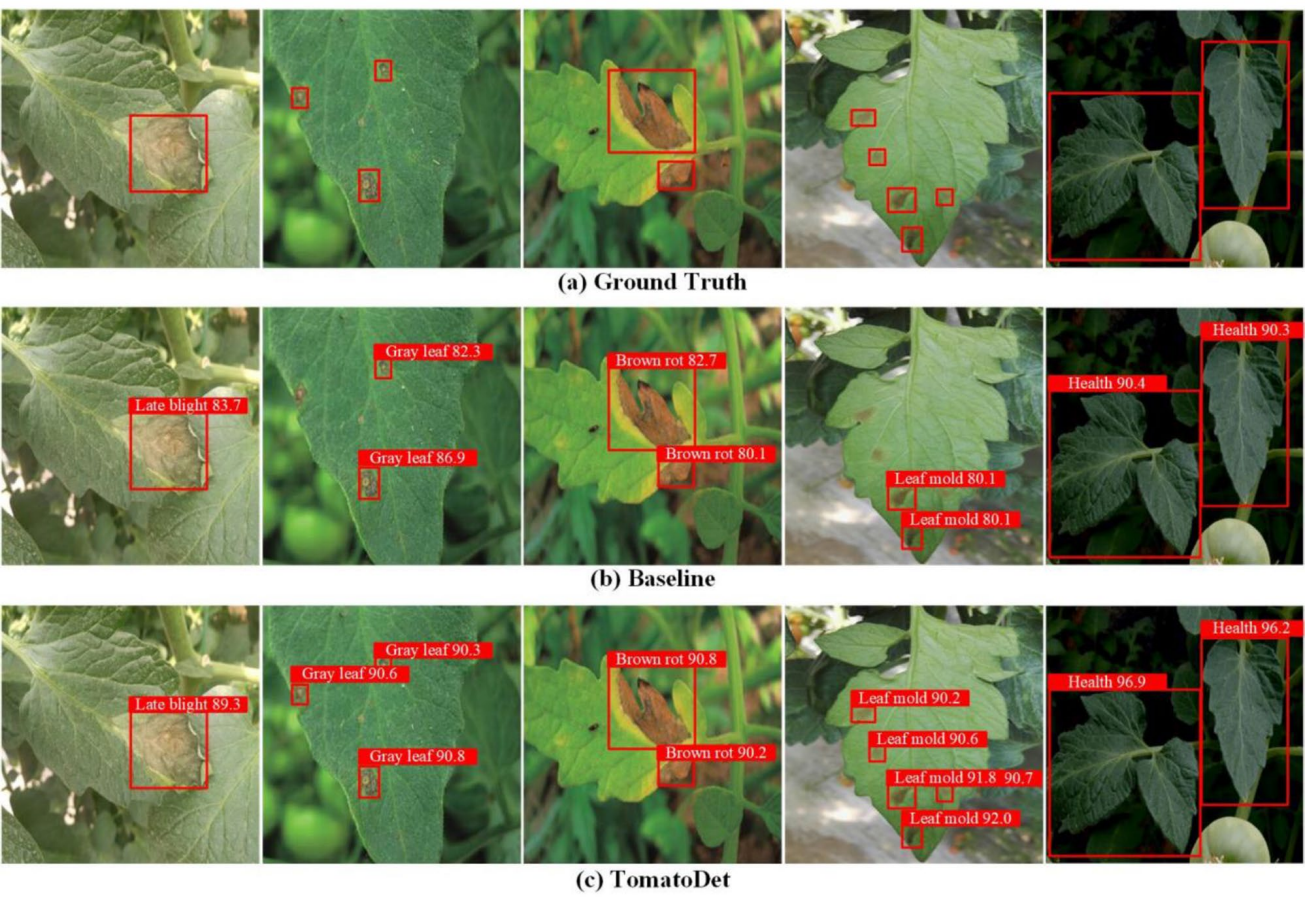
Comparison of the detection efficacy of TomatoDet and the baseline model

The proposed TomatoDet shows a significant improvement regarding the accuracy of disease detection in tomatoes compared to the original model, as depicted in Fig. 19.

Figure 19 visually demonstrates the advantages of the proposed TomatoDet through comparative visualization. As depicted in Fig. 19, the baseline model suffers from missed detections and inaccurate localization for diseases and has difficulties detecting small targets. The proposed TomatoDet detected some diseases that the original model failed to detect. The added attention mechanism effectively suppresses the interference of background information, making the localization more accurate. The proposed feature fusion module enhances the detection ability for small targets, resulting in higher detection accuracy and better model robustness. The overall disease recognition results show that the proposed TomatoDet has better global information extraction capabilities compared to the baseline model and performs better in identifying diseases with dark and blurry brightness, indicating a stronger generalization ability than the original model. Therefore, the proposed TomatoDet presents more promising results, making it a potential solution for plant disease object detection.

## Conclusion

In this study, we introduce TomatoDet, a real-time algorithm designed for identifying tomato diseases utilizing the proposed Swin-DDETR, Meta-ACON and IBiFPN to enhance performance. Our proposed TomatoDet achieves precise real-time detection of tomato diseases by optimizing the backbone network, activation functions, and feature fusion structure. The efficacy of TomatoDet is demonstrated through experimental results, showcasing improved detection accuracy for tomato diseases. It outperforms mainstream disease detection algorithms, achieving a mean Average Precision (mAP) of 92.3% on our self-built tomat disease dataset. Moreover, our algorithm attains a frame rate of 46.6 frames per second (FPS) on the Tesla T4, meeting the demands for real-time detection of tomato diseases in greenhouse environments.

While this study has made certain achievements in tomato disease detection, there is still much research to be conducted before transitioning from the experimental stage to practical application, thus truly assisting tomato growers. Future endeavors will mainly include:

1. Greenhouse environments significantly influence model performance. Expanding the dataset of real-world tomato disease samples and implementing a model’s autonomous continuous learning strategy will enhance disease detection accuracy in greenhouse settings.
2. Subsequent research will delve into the early occurrence patterns of typical high-incidence diseases, enabling timely detection and prevention of early-stage diseases.
3. Future studies will integrate the tomato disease spraying robot developed by our team, transmitting disease occurrence location information to the spraying robot for targeted spraying operations. Subsequently, field evaluations of the robot’s operation performance will be conducted by professional crop protection personnel. Finally, based on operational data, inspection processes and performance will be optimized, accelerating the production and implementation of tomato disease inspection robots through statutory third-party performance testing.

## Data Availability

The data utilized in this paper is obtained through self-gathering and is made publicly available (a part of it) to make the study reproducible. It can be accessed at https://github.com/tyuiouio/plant-disease-detection-in-real-field. If you want to request the complete dataset and code, please email the corresponding author.
